# Effects of a pain oriented biobehavioral therapeutic education program on brain plasticity and pain intensity in subjects with chronic musculoskeletal pain: a feasibility study of a randomized controlled trial

**DOI:** 10.3389/fnins.2025.1664158

**Published:** 2025-11-17

**Authors:** Silvia Di-Bonaventura, Aser Donado-Bermejo, Luis Matesanz-García, Miguel Molina-Álvarez, José Vicente León-Hernández, Ángel Lizcano-Álvarez, Sergio Lerma-Lara, Maximiliano Nogales-Morales, Nuria Molina, Josué Fernández-Carnero, Francisco Gurdiel-Álvarez, Raúl Ferrer-Peña

**Affiliations:** 1 Department of Physical Therapy, Occupational Therapy, Rehabilitation and Physical Medicine, Rey Juan Carlos University, Alcorcón, Spain; 2 Escuela Internacional de Doctorado, Faculty of Health Sciences, Rey Juan Carlos University Alcorcón, Alcorcón, Spain; 3 Cognitive Neuroscience, Pain and Rehabilitation Research Group (NECODOR), Faculty of Health Sciences, Rey Juan Carlos University, Madrid, Spain; 4 Department of Physiotherapy, Centro Superior de Estudios Universitarios La Salle, Universidad Autónoma de Madrid, Madrid, Spain; 5 CranioSPain Research Group, Centro Superior de Estudios Universitarios La Salle, Universidad Autónoma de Madrid, Madrid, Spain; 6 Area of Pharmacology, Nutrition and Bromatology, Department of Basic Health Sciences, Unidad Asociada I+D+i Instituto de Química Médica (IQM) CSIC-URJC, Rey Juan Carlos University, Alcorcón, Spain; 7 Motion in Brains Research Group, Institute of Neuroscience and Sciences of the Movement (INCIMOV), Centro Superior de Estudios Universitarios La Salle, Universidad Autónoma de Madrid, Madrid, Spain; 8 Department of Nursing and Stomatology, Faculty of Health Sciences, Universidad Rey Juan Carlos, Alcorcón, Madrid, Spain; 9 Instituto de Rehabilitación Funcional La Salle, Aravaca, Calle Ganímedes, Madrid, Spain; 10 Independent Researcher, Madrid, Spain; 11 Grupo Multidisciplinar de Investigación y Tratamiento del Dolor, Grupo de Excelencia Investigadora URJC-Banco de Santander, Alcorcón, Spain; 12 La Paz Hospital Institute for Health Research, IdiPAZ, Madrid, Spain; 13 Musculoskeletal Pain and Motor Control Research Group, Faculty of Sport Sciences, Universidad Europea de Madrid, Villaviciosa de Odón, Spain; 14 Grupo de Investigación Clínico-Docente sobre Ciencias de la Rehabilitación (INDOCLIN), CSEU La Salle, UAM, Madrid, Spain

**Keywords:** pain education, brain plasticity, chronic pain, musculoskeletal pain, BDNF

## Abstract

**Background:**

Chronic pain significantly impacts the physical, emotional, and social wellbeing of individuals. Despite advances in treatments, chronic pain prevalence continues to rise, emphasizing the need for comprehensive therapeutic approaches.

**Objective:**

This study aimed to evaluate the feasibility and preliminary effects of a one-month Pain Oriented Biobehavioral Therapeutic Education (POBTE) program on clinical outcomes for chronic primary musculoskeletal pain.

**Methods:**

In a single-blind feasibility pilot of a randomized controlled trial, 16 participants were assigned to an intervention group receiving POBTE education and exercise (*n =* 8) or a control group (*n =* 8) participating in exercise only. Primary outcomes were pain intensity, measured by the Numeric Pain Rating Scale, and Brain-Derived Neurotrophic Factor (BDNF) plasma levels.

**Results:**

The intervention group showed a significant increase in BDNF levels from a mean of 2.174 at baseline to 3.063 at the end of treatment (*p* = 0.001, r = 0.63), with a non-significant reduction in pain intensity. Secondary outcomes, including anxiety, sleep quality, and physical activity, improved significantly. The results, however, should be interpreted cautiously due to the small sample size.

**Conclusion:**

The POBTE program appears feasible and acceptable, showing preliminary signals consistent with potential improvements in several clinical variables related to chronic pain management. These exploratory findings support the need for larger-scale, adequately powered trials.

## Background

Chronic pain, defined as pain that persists for more than 3 months (Treede et al., 2015), represents a considerable public health problem (Goldberg and McGee, 2011). Its impact on patients’ quality of life is profound, affecting their physical (Hadi et al., 2019), emotional (Burke et al., 2015), and social wellbeing (Dueñas et al., 2016). Despite ongoing advancements in treatment research, the prevalence of chronic pain continues to rise (Nahin et al., 2023), highlighting the need for therapeutic approaches that not only address pain per se but also consider its long-term effects across different dimensions in a comprehensive approach to the affected individual (Fine, n.d.; Hylands-White et al., 2017).

In the treatment of chronic pain, Pain Neuroscience Education (PNE) has been identified as a key strategy (Lepri et al., 2023; Louw et al., 2011). Traditionally, many educational interventions have primarily focused on cognitive aspects, providing information about the biological and physiological bases of pain (Louw et al., 2016; Moseley et al., 2015). However, education that is limited to cognitive aspects may not be sufficient to fully address the needs of patients with chronic pain (Oosterhaven et al., 2023). A purely cognitive approach often omits the incorporation of essential behavioral elements necessary for a multidimensional management of pain (Arlinghaus and Johnston, 2018). Behavioral changes, for example, are crucial for the effective implementation of pain management strategies in patients’ daily lives (Cuenca-Martínez et al., 2022). Additionally, biological aspects such as changes in brain plasticity and associated clinical variables can provide valuable insights into the effects of educational interventions at the neurophysiological level.

Therefore, a broader approach to pain education might include not only the transmission of information but also the promotion of practical skills and techniques that encourage behavioral changes. Moreover, integrating the assessment of biological indicators of these changes, such as Brain-Derived Neurotrophic Factor (BDNF), which has been linked to brain plasticity (Garraway, 2023) and pain modulation (Merighi, 2024), could offer a deeper understanding of how interventions can directly influence brain physiology.

The present study was designed to evaluate the feasibility of a one-month, specific pain education program for the treatment of chronic primary pain. The main objective was to determine whether the study procedures and intervention could be implemented as planned, while also generating preliminary data on clinical outcomes and neuroplasticity markers. The results of this pilot feasibility randomized trial are expected to provide insights into both the viability and potential effectiveness of the program, thereby offering a foundation for future research into treatment methodologies for chronic pain.

## Methods

### Trial design

This study was conceived as a pilot feasibility randomized clinical trial with a single-blind, parallel-group design, registered at ClinicalTrials.gov (NCT05623579) and approved under the internal ethical committee number of Rey Juan Carlos University (1901202202822). All the procedure adhered strictly to established protocols according to the CONSORT Statement criteria to ensure study quality and transparency (Eldridge et al., 2016). Additionally, the Tidier Checklist criteria (Hoffmann et al., 2014) for education was applied, ensuring the completeness and consistency of the information collected during the educational process. Given the pilot nature of this trial, the primary aim was not to test efficacy but to evaluate feasibility and acceptability of the protocol in preparation for a larger confirmatory trial.

### Participants

Participant recruitment was conducted through the distribution of flyers in private clinics within the Community of Madrid and via social media platforms. The recruitment period lasted 3 months (January–March 2023). Patients aged between 18 and 65 years with primary musculoskeletal chronic pain (including chronic primary cervical, thoracic, back, or limb pain) according to ICD-11 (Nicholas et al., 2019) and lasting at least 3 months were included. Participants were required not to have received physiotherapeutic treatment for the same condition in the last 6 months. Additionally, patients needed to have the ability to perform all clinical tests and understand the study process, providing their informed consent. Exclusion criteria included the presence of systemic, neurological, oncological, or inflammatory diseases, type II diabetes, psychiatric pathologies, and pregnancy. At baseline, participants were specifically asked about chronic medication use; none of them reported taking long-term pharmacological treatments. They were also instructed to report the use of any analgesics or other medication during the intervention; however, no such cases occurred in our sample.

### Intervention

*POBTE Group (Intervention):* Patients in the intervention group participated in a therapeutic education program called POBTE (Pain Oriented Biobehavioral Therapeutic Education), combined with exercise. Pain education, based on the previously published POBTE protocol (Di Bonaventura et al., 2024), was conducted twice a week in 40-min sessions over a period of 4 weeks. The structure and content of each education session were predefined and are detailed in Supplementary Figure 1, which specifies day by day the topics covered (e.g., physiological mechanisms of pain, pain modulation mechanisms, cognitive-behavioral strategies, and self-efficacy techniques).

Additionally, following the education, patients engaged in supervised exercise sessions twice a week, each lasting 30 min, which included 5 min of warm-up (joint mobility and light walking), 20 min of a progressive strength and aerobic circuit (e.g., squats, push-ups, elastic-band exercises, step, brisk walking in place), and 5 min of cool-down (stretching of major muscle groups and breathing exercises). The exercise program was designed to be progressive and adapted to the individual capacities of each patient. Dosing was based on a light-moderate effort on the Borg scale (Stamford, 1976), starting at an RPE of 3/10 in the first week and progressively increasing to 6/10 by the final week. This exercise protocol was standardized and applied identically in both study groups, with adaptations made only in load or repetitions according to participant capacity. Treatment fidelity was ensured by (i) using a structured manual for both education and exercise, (ii) training the therapists before the trial began, and (iii) systematically recording session attendance and adherence. The program was delivered by two physiotherapists with 7 years of clinical experience in chronic pain management and specific training in therapeutic pain education and biobehavioral interventions.

Active Control Group: Patients in the control group participated in the same structured exercise program, with the same frequency and intensity as the intervention group. This ensured that the only difference between groups was the addition of the POBTE education sessions in the intervention group. To ensure fidelity, control group sessions also followed the same exercise protocol and adherence was monitored in the same way as for the intervention group.

### Outcomes

Given the pilot feasibility nature of this trial, outcome measures were collected primarily to describe preliminary trends and variability. These data were intended to support planning and sample size estimation for a future confirmatory trial, rather than to provide definitive evidence of efficacy.

The following measurements were taken in the subjects at baseline, half treatment (15 days), at the end of the program, and 1 month after completing the program.

### Sociodemographic

Sex, Age, body mass index (BMI), Marital status (Single, Married, Divorced, Widowed), Employment status (Employed, Unemployed, Retired, Sick Leave, Student, Housekeeper, Other), Study level (None, Primary, Secondary, University).

### Primary outcomes

*Pain Intensity:* Pain intensity was measured using a 100 mm Numeric Pain Rating Scale (NPRS), where 0 represents “no pain” and 100 represents the “worst pain imaginable.” Participants marked a point on the line that best reflected the pain they were experiencing at the time of measurement. Higher scores indicated higher levels of pain, and the administration required less than 1 min (Hjermstad et al., 2011). This variable was assessed only in chronic primary musculoskeletal pain and widespread pain patients. The Minimal Clinically Important Difference (MCID) for the NPRS in musculoskeletal pain is generally considered to be a reduction of 2 points or 30% from baseline values (Farrar et al., 2001), which was taken into account when interpreting changes.

*BDNF Plasma Levels:* Blood samples were collected in EDTA-anticoagulant tubes and centrifuged at 1000 × g for 15 min within 30 min of collection. The separated plasma was aliquoted into 2 mL Eppendorf tubes and stored at −80°C until analysis. For the analysis, human BDNF ELISA kits (Abbexa, Catalog No: abx150799, Cambridge, United Kingdom) were used according to the manufacturer’s protocol. The enzymatic reaction was stopped with a stop solution, and the optical density was measured at 450 nm using a FLUOstar Omega microplate reader (BMG LABTECH, Offenburg, Germany). BDNF concentrations were calculated using a standard curve generated with the standard solutions provided in the kit. According to the manufacturer, the assay performance included a detection range of 12.5–800 pg./mL, a sensitivity of ~20 pg./mL, an intra-assay CV < 8%, and an inter-assay CV < 10%. In addition, for each participant, one control well was systematically included in every assay plate to ensure consistency of measurements. To control for circadian variation, all blood samples were collected between 10:00 and 12:00 a.m. after an overnight fast. Participants were also instructed to refrain from performing intense physical exercise during the 24 h prior to blood collection, as this may acutely alter BDNF levels. In addition, the temperature of the education and exercise rooms was kept constant at 21°C throughout the intervention to ensure standardized environmental conditions. Although no established MCID exists for BDNF, variability estimates from this pilot were used to inform the sample size calculation for a future definitive trial.

### Secondary outcomes

*Anxiety and Depression:* Assessed using the validated Spanish version of the Hospital Anxiety and Depression Scale (HADS), which is divided into two subscales of 7 items each: 1) Depression (HADS-Dep); and 2) Anxiety (HADS-Anx). The subscales of HADS showed internal consistency indices recommended for screening tools. The items in HADS demonstrated a positive correlation with the total score of the anxiety and depression subscales. HADS was found to perform well in assessing the symptom severity and caseness of anxiety disorders and depression in both somatic, psychiatric, and primary care patients and in the general population (Bjelland et al., n.d.).

*Quality of Life:* Measured with the EuroQoL-5D (EQ-5D) questionnaire, a self-report instrument for assessing health-related quality of life. It comprises three elements: a descriptive scale of 5 factors, a second element composed of a vertical NRS, and a social value index generated by the instrument. The EQ-5D has shown good psychometric properties. This variable was assessed only in chronic primary musculoskeletal pain and widespread pain patients (Rabin and De Charro, 2001).

*Pain Catastrophizing:* Assessed using the Spanish version of the Pain Catastrophizing Scale (PCS), which has demonstrated adequate psychometric properties for evaluating this construct and a high internal consistency (Cronbach’s alpha of 0.92 (95% CI 0.91–0.93)) (Osman et al., 2000).

*Kinesiophobia:* Measured using the Tampa Scale for Kinesiophobia (TSK), a self-report questionnaire comprising 11 items. The internal consistency of the TSK is high, with Cronbach’s alpha coefficients ranging from 0.74 to 0.93, indicating strong reliability. Test–retest reliability is also good, with correlation coefficients ranging from 0.75 to 0.88(Woby et al., 2005).

*Sleep quality:* Assessed using the Pittsburgh Sleep Quality Index (PSQI), a self-rated questionnaire consisting of 19 items categorized into seven components: subjective sleep quality, sleep latency, sleep duration, habitual sleep efficiency, sleep disturbances, use of sleep medication, and daytime dysfunction. The internal consistency of the PSQI is high, with Cronbach’s alpha coefficients ranging from 0.77 to 0.83, indicating strong reliability. Test–retest reliability is also good, with correlation coefficients ranging from 0.85 to 0.87(Larche et al., 2021).

*Physical activity levels:* Measured using the International Physical Activity Questionnaire (IPAQ), a self-report questionnaire of 27 items designed to assess the frequency, duration, and intensity of physical activity. The internal consistency of the IPAQ is moderate to high, with Cronbach’s alpha coefficients ranging from 0.73 to 0.95. Test–retest reliability is good, with correlation coefficients ranging from 0.70 to 0.88 (Craig et al., 2003).

### Sample size estimation for full trial

The pilot sample size (*n =* 16) was pragmatically chosen to evaluate feasibility, while also allowing the collection of preliminary data on clinical and biological outcomes. To estimate the sample size needed for the full trial, plasma BDNF levels, measured using an ELISA Kit, were used as the primary outcome measure. We analyzed the variability in BDNF measurements and calculated the differences between the control and intervention groups over time, as such data were not available from previous studies. This approach also provided a more precise estimate of the likely dropout rate in a larger trial, which is an essential feasibility outcome. The G*Power software (version 3.1) was used with the following parameters: estimated effect size *f* = 0.40, significance level *α* = 0.05, statistical power of 80%, assuming a correlation between measurements of 0.5 and perfect sphericity (*ε* = 1.0). The analysis indicated that 51 participants per group would be required, representing a total of 102 subjects, to detect statistically significant between-group differences over time in a future confirmatory trial.

### Randomization and allocation

**Figure 1:**
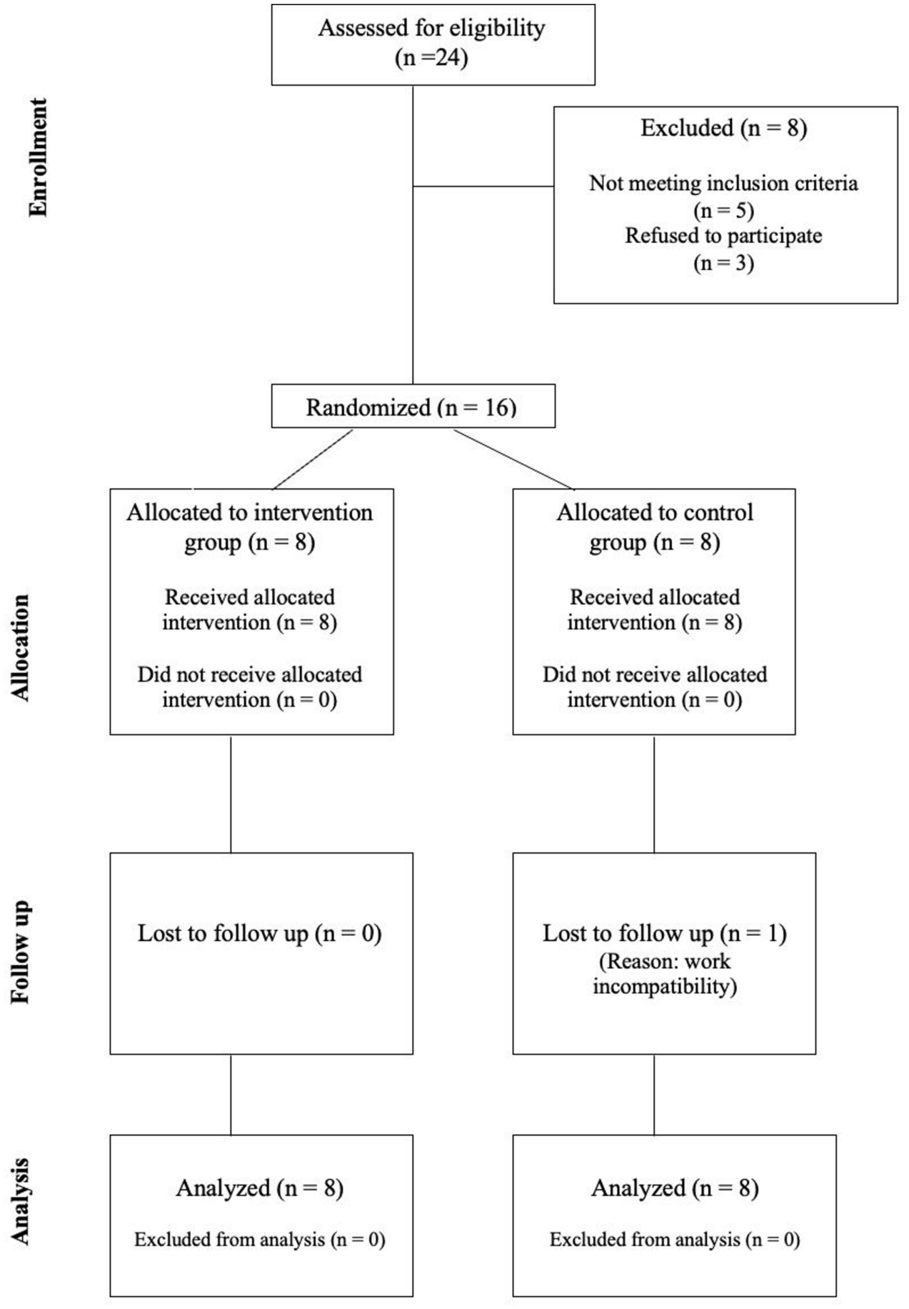
CONSORT flow diagram.

The allocation of participants was conducted using computer-generated random numbers created in SPSS (IBM SPSS Statistics, version 31) to ensure an equitable distribution between the intervention and control groups. Participants were randomized in a 1:1 ratio. The randomization sequence was prepared in advance by an independent researcher not involved in recruitment, assessment, or intervention delivery. No blocking or stratification was applied given the small sample size. Allocation concealment was ensured using a pre-generated allocation list sequentially numbered and placed in opaque, sealed envelopes. These envelopes were stored securely and opened sequentially by another researcher only after the participant had provided informed consent and completed the initial assessment. This procedure ensured that investigators enrolling participants and collecting baseline data had no prior knowledge of the upcoming allocation. Figure 1 and Supplementary Figure 2 show the flow of participants through the study.

### Blinding

In the study, blinding was specifically implemented for the statistician, those responsible for the randomization and allocation of participants, and those administering the questionnaires, in order to minimize bias in data analysis and collection. Due to the nature of the interventions, blinding of participants and therapists delivering the education and exercise programs was not possible. To mitigate potential bias, therapists followed a standardized intervention protocol, and participants were instructed not to disclose their allocation during assessments. Outcome assessors collected data using standardized scripts and had no involvement in intervention delivery. Finally, statistical analyses were conducted using coded datasets, with groups labeled generically until the analysis was completed.

## Statistical methods

All analyses were conducted using the SPSS statistical software (version 29). A *p*-value of less than 0.05 was considered statistically significant. The normality of the data was assessed using the Shapiro–Wilk test. Since none of the study variables followed a normal distribution, non-parametric tests were employed, in line with the sample size and the nature of the data.

Descriptive variables are presented as frequencies and percentages, n (%), for qualitative variables, and as medians and interquartile ranges [IQR] for quantitative variables. All confidence intervals were calculated using bootstrap (10,000 resamples). To compare repeated measurements at different time points (Pre, Intermediate, Post, and Follow-up) within each group (Intervention and Control), the Friedman test was used. Subsequently, *post hoc* comparisons were performed using the Wilcoxon test to evaluate differences between the various time points within each group. Additionally, to compare the intervention and control groups at each time point, the Mann–Whitney U test was employed. The effect size was calculated using Rosenthal’s r, which was interpreted according to standard criteria: 0.1 indicates a small effect, 0.3 a moderate effect, and 0.5 a large effect. In light of the exploratory nature of this pilot and the limited sample size, corrections for multiple testing were not applied, as they could disproportionately increase the likelihood of type II errors. This approach aligns with the CONSORT extension for pilot and feasibility trials, which highlights that statistical testing in this context serves primarily to provide preliminary estimates to inform the design of future definitive RCTs (Eldridge et al., 2016). Confidence intervals are therefore reported descriptively to illustrate variability rather than to draw population-level conclusions. Finally, considering the repeated measures design with both inter-subject (group) and intra-subject (time) factors, a sample size calculation was performed to estimate the requirements for a future larger-scale clinical trial using a two-group mixed ANOVA with four measurements.

## Results

The study included a total of 16 subjects, evenly distributed between two groups: intervention (*n =* 8) and control (*n =* 8). The initial sociodemographic variables, including sex, marital status, employment status, education level, and the duration of the condition, showed no statistically significant differences between the two groups, with *p*-values greater than 0.05 in all comparisons. This suggests that the groups were comparable in terms of baseline characteristics before the intervention. The characteristics of participants at baseline are shown in Table 1 and Supplementary Table 1.

**Table 1:** Descriptive sociodemographic variables.

Variable	Category	Intervention (*n =* 8)	Control (*n =* 8)	*p*-value	Effect size (*r*)
Sex	Men	4 (50%)	5 (62.5%)	0.614	0.13
	Women	4 (50%)	3 (37.5%)		
Marital Status	Single	2 (25%)	2 (25%)	1.000	0.00
	Married	6 (75%)	6 (75%)		
Employment Status	Employed	7 (87.5%)	5 (62.5%)	0.506	0.38
	Unemployed	1 (12.5%)	1 (12.5%)		
	Retired	0 (0%)	1 (12.5%)		
	Housework	0 (0%)	0 (0%)		
	Student	0 (0%)	0 (0%)		
	Sick Leave	0 (0%)	1 (12.5%)		
Study Level	Secondary	3 (37.5%)	5 (62.5%)	0.317	0.25
	University	5 (62.5%)	3 (37.5%)		
BMI		23.05 [19.80–25.00]	28.80 [25.30–35.48]	0.869	0.04
Pain Evolution Time (months)		54.0 [30.0–160.5]	66.0 [24.0–237.0]	0.850	0.05
Plasma BDNF (ng/ml)		2.174 [2.035–2.484]	2.200 [2.027–2.368]	0.609	0.12
NPRS (0–10)		6.0 [4.25–6.5]	5.75 [3.625–9.0]	0.106	0.43
EQ-5D-5L Health VAS (0–100)		80.0 [68.75–83.75]	67.5 [32.5–78.75]	0.196	0.35
HADS Anxiety (0–14)		7.0 [4.5–9.75]	9.0 [6.0–13.75]	0.250	0.30
HADs Depression (0–14)		3.5 [3.0–5.75]	8.0 [3.0–10.75]	0.176	0.38
Pain Catastrophizing Scale (0–52)		11.5 [5.5–23.5]	16.0 [12.25–34.25]	0.374	0.22
Tampa Scale of Kinesiophobia (11–44)		22.0 [19.25–28.5]	28.5 [23.5–33.0]	0.926	0.02
Pittsburg Sleep Quality Index		9.5 [7.25–13.75]	13.5 [9.75–15.75]	0.850	0.05
IPAQ (Mets)		2433.5 [1461.0–4109.3]	3052.0 [396.0–5784.8]	0.609	0.12

Plasma BDNF concentrations are expressed in ng/mL. Pre-analytic controls included overnight fasting, standardized collection between 10:00 and 12:00 h, processing within 30 min of extraction, and storage at −80°C.

**Figure 2:**
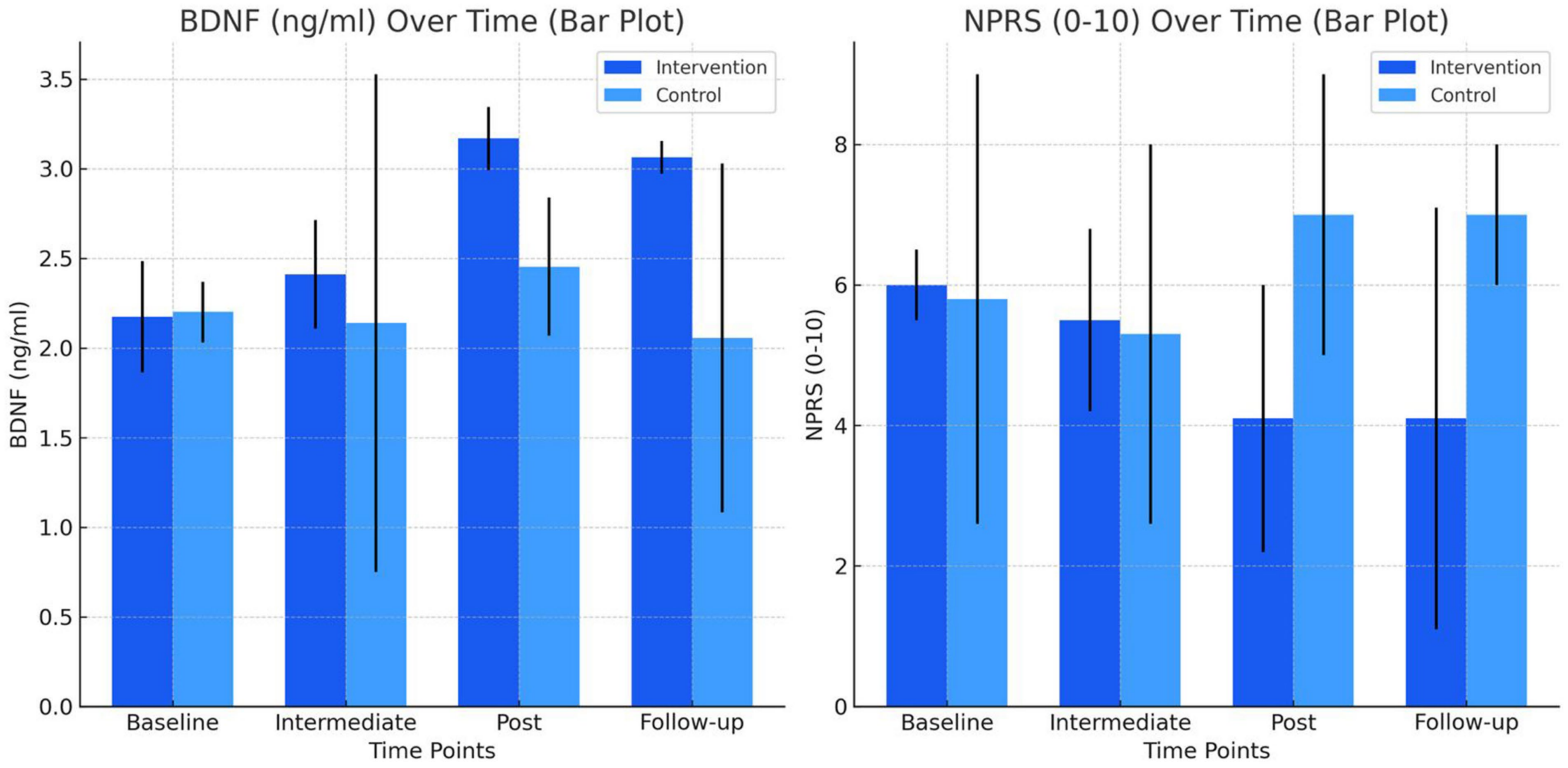
Barplot primary outcomes.

Regarding the primary variables (Table 2), BDNF levels in the intervention group showed a significant increase from a mean of 2.174 (95% CI: 2.035–2.484) at baseline to a mean of 3.063 (95% CI: 2.856–3.154) at follow-up, with statistically significant increases both post-treatment (*p* = 0.001*, r = 0.63) and at follow-up (*p* = 0.049*, r = 0.51). In contrast, the control group maintained relatively stable BDNF levels, starting with a mean of 2.200 (95% CI: 2.027–2.368) and ending at 2.056 (95% CI: 1.993–3.029) without significant changes (Figure 2).

**Table 2:** Multiple comparison of primary outcomes.

Variables	Baseline	Intermediate	Post	Follow-up	ΔMedian (Final-Pre); IC 95%
BDNF (ng/ml)					
Intervention	2.174 [2.035–2.484]	2.410 [2.228–2.713]	3.168 [2.850–3.344]	3.063 [2.856–3.154]	ΔMed = 0.03; IC 95% = [−0.82, 1.09]
Control	2.200 [2.027–2.368]	2.139 [1.993–3.527]	2.453 [2.339–2.838]	2.056 [1.993–3.029]	ΔMed = −1.90; IC 95% = [−0.63, 0.79]
Difference in medians; Hodges-Lehman estimate *δ*	ΔMed = − 0.25; δ = 0.25	ΔMed = − 0.25; δ = 0.25	ΔMed = − 0.25; δ = 0.25	ΔMed = − 0.25; δ = 0.25	
NPRS (0–10)					
Intervention	6.0 [4.3–6.5]	5.5 [4.0–6.8]	4.1 [2.3–6.0]	4.1 [2.4–7.1]	ΔMed = − 0.50; IC 95% = [−2.00, 1.50]
Control	5.8 [3.6–9.0]	5.3 [2.3–8.0]	7.0 [3.0–9.0]	7.0 [3.0–8.0]	ΔMed = −1.90; IC 95% = [−4.00,1.00]
Difference in medians; Hodges-Lehman estimate δ	ΔMed = − 0.25; δ = 0.25	ΔMed = 1.50; δ = 0.50	ΔMed = 2.90; δ = 1.25	ΔMed = 0.416; δ = 0.21	

BDNF, Brain Derived Neurotrophic Factor; NPRS, Numeric Pain Rating Scale. Plasma BDNF concentrations are expressed in ng/mL. Pre-analytic controls included overnight fasting, standardized collection between 10:00–12:00 h, processing within 30 min of extraction, and storage at −80°C.

The analysis between groups revealed statistically significant differences in BDNF levels at the end of treatment and at follow-up. In the post-treatment evaluation, the intervention group had a median of 3,168 ng/mL (interquartile range: 2,850–3,344), significantly higher than the control group, with a median of 2,453 ng/mL (interquartile range: 2,339–2,838) (*p* = 0.015; r = 0.63). This difference was maintained in the follow-up evaluation, where the intervention group showed a median of 3,063 ng/mL (interquartile range: 2,856–3,154), compared to 2,056 ng/mL (interquartile range: 1,993–3,029) in the control group (*p* = 0.049; r = 0.51).

As for pain intensity, the intervention group showed a reduction in scores from an initial mean of 6.0 (95% CI: 4.3–6.5) to 4.1 (95% CI: 2.4–7.1) at follow-up, although this reduction was not statistically significant (*p* = 0.459). The control group showed minor fluctuations in NPRS, starting at 5.8 (95% CI: 3.6–9.0) and increasing to 7.0 (95% CI: 3.0–9.0) at follow-up, with no statistically significant differences (Figure 2).

In terms of secondary variables (Table 3), the study revealed that anxiety scores in the intervention group decreased significantly from 7.0 (95% CI: 4.5–9.8) to 4.5 (95% CI: 3.0–6.8) at follow-up (*p* = 0.008*), while the control group showed no significant changes, with an initial score of 9.0 (95% CI: 6.0–13.8) and a final score of 7.0 (95% CI: 4.0–12.0) (*p* = 0.057). Similarly, depression scores in the intervention group showed a significant reduction from 3.5 (95% CI: 3.0–5.75) to 2.5 (95% CI: 1.25–4.75) (*p* = 0.017*), while the control group’s scores remained constant without significant changes.

**Table 3:** Multiple comparison of secondary outcomes.

Variables	Baseline	Intermediate	Post	Follow-up	ΔMedian (Final-Pre); IC 95%
HADS anxiety					
Intervention	7.0 [4.5–9.8]	6.0 [4.3–9.8]	5.5 [2.3–7.8]	4.5 [3.0–6.8]	ΔMed = −3.00; IC 95% = [−4.00,1.00]
Control	9.0 [6.0–13.8]	9.0 [6.0–13.0]	7.0 [4.0–12.0]	7.0 [4.0–12.0]	ΔMed = −1.50; IC 95% = [−4.00, 0.00]
Difference in medians; Hodges-Lehman estimate δ	ΔMed = 2.00; δ = 2.5	ΔMed = 3.00; δ = 3.00	ΔMed = 1.50; δ = 4.00	ΔMed = 2.50; δ = 3.00	
HAD depression					
Intervention	3.5 [3.0–5.75]	3.5 [1.0–6.0]	4.0 [0.5–5.75]	2.5 [1.25–4.75]	0 ΔMed = −1.00; IC 95% = [−3.00, 3.00]
Control	8.0 [3.0–10.75]	8.0 [4.0–10.0]	8.0 [5.0–10.0]	7.0 [3.0–10.0]	ΔMed = 0.50; IC 95% = [−2.00, 1.00]
Difference in medians; Hodges-Lehman estimate δ	ΔMed = 4.50; δ = 4.00	ΔMed = 4.50; δ = 4.00	ΔMed = 4.00; δ = 5.00	ΔMed = 4.50; δ = 3.00	
PCS					
Intervention	11.5 [5.5–23.5]	13.5 [7.5–22.3]	10.5 [4.3–17.0]	8.5 [2.3–15.5]	ΔMed = −1.00; IC 95% = [−9.00, 4.00]
Control	16.0 [12.3–34.3]	13.0 [8.0–33.0]	13.0 [8.0–28.5]	13.0 [0.0–24.0]	ΔMed = − 1.00; IC 95% = [−6.50, 4.00]
Difference in medians; Hodges-Lehman estimate δ	ΔMed = 4.50; δ = 7.00	ΔMed = −0.50; δ = 2.50	ΔMed = 2.00; δ = 1.00	ΔMed = 4.50; δ = 6.00	
TSK					
Intervention	22.0 [19.25–28.5]	20.5 [16.0–27.0]	19.0 [17.0–24.0]	24.0 [18.25–26.75]	ΔMed = − 6.00; IC 95% = [−7.00, 2.00]
Control	28.5 [23.5–33.0]	33.0 [21.0–37.0]	26.0 [22.0–34.0]	28.0 [19.0–34.0]	ΔMed = − 3.00; IC 95% = −9.00, 1.00]
Difference in medians; Hodges-Lehman estimate δ	ΔMed = 6.50; δ = 5.00	ΔMed = 12.50; δ = 6.50	ΔMed = 7.00; δ = 7.00	ΔMed = 4.50; δ = 5.50	
PSIQ					
Intervention	9.5 [7.3–13.8]	8.0 [6.5–11.3]	6.5 [6.0–9.0]	7.5 [6.3–9.8]	ΔMed = − 1.00; IC 95% = [−8.00, 1.00]
Control	13.5 [9.8–15.8]	13.0 [10.0–16.0]	12.0 [10.0–13.0]	15.0 [14.0–16.0]	ΔMed = − 0.50; IC 95% = [−6.50, 1.00]
Difference in medians; Hodges-Lehman estimate δ	ΔMed = 4.00; δ = 3.00	ΔMed = 2.00; δ = 2.00	ΔMed = 6.00; δ = 5.50	ΔMed = 0.00; δ = 2.50	
IPAQ					
Intervention	2433.5 [1461.0–4109.3]	2529.0 [2121.5–4639.3]	4486.5 [3630.8–4946.3]	2466.0 [1392.8–4041.8]	ΔMed = −863.00; IC 95% = [−1425.00, 1187.00]
Control	3052.0 [396.0–5784.8]	2358.0 [1428.0–2856.0]	1908.0 [0.0–2820.0]	1733.0 [1116.0–2772.0]	ΔMed = 2053.00; IC 95% = [−574.00, 3009.00]
Difference in medians; Hodges-Lehman estimate δ	ΔMed = 618.00; δ = 1.50	ΔMed = −171.00; δ = −777.50	ΔMed = −2578.00; δ = −2507.00	ΔMed = −733.00; δ = −861.00	
EQ-5D-5L Health VAS					
Intervention	80.0 [68.8–83.8]	82.5 [71.3–88.8]	85.0 [72.5–88.8]	72.5 [61.3–85.0]	ΔMed = −15.00; IC 95% = [−50.00, 50.00]
Control	67.5 [32.5–78.8]	60.0 [25.0–80.0]	50.0 [15.0–80.0]	70.0 [30.0–75.0]	ΔMed = 5.00; IC 95% = [−15.00, 20.00]
Difference in medians; Hodges-Lehman estimate δ	ΔMed = −12.50; δ = −10.00	ΔMed = −22.50; δ = −22.50	ΔMed = −35.00; δ = −35.00	ΔMed = −2.50; δ = −10.00	

HAD, Hospital Anxiety and Depression Scale; PCS, Pain Catastrophizing Scale; TSK, Tampa Scale of Kinesiophobia; PSQI, Pittsburgh Sleep Quality Index; IPAQ, International Physical Activity Questionnaire; EQ-5D-5L Health VAS, EuroQoL 5 Dimensions 5 Levels Health Visual Analogue Scale.

Regarding PCS and TSK, neither group showed statistically significant changes throughout the study. PCS scores in the intervention group remained relatively stable, while those in the control group fluctuated without reaching statistical significance. Although the intervention group showed a slight decrease in TSK, it was not significant (from 22.0 to 19.0), while the control group experienced a slight, non-significant increase.

On the other hand, the intervention group experienced significant improvements in sleep quality, with a reduction in the mean score from 9.5 (95% CI: 7.3–13.8) to 7.5 (95% CI: 6.3–9.8) at follow-up (*p* = 0.002*). The control group, however, did not show significant improvements in this aspect. At follow-up, the intervention group obtained significantly lower scores (median: 7.5; interquartile range: 6.3–9.8) compared to the control group (median: 15.0; interquartile range: 14.0–16.0) (*p* = 0.002; r = 0.73), which indicates a clinically relevant improvement in the intervention group. The observed improvement in the overall PSQI score in the intervention group exceeded the minimum clinically important difference of 4.4 points, indicating a significant improvement in sleep quality.

Additionally, physical activity levels showed a significant increase in the intervention group, rising from 2433.5 MET-minutes/week (95% CI: 1461.0–4109.3) to 4486.5 MET-minutes/week (95% CI: 3630.8–4946.3) at follow-up (*p* = 0.024*). In contrast, the control group did not show significant changes in physical activity. Regarding between-groups differences, physical activity levels were significantly higher in the intervention group after the intervention (median: 4486.5 MET-minutes/week; interquartile range: 3630.8–4946.3) than in the control group (median: 1908.0 MET-minutes/week; interquartile range: 0.0–2820.0) (*p* = 0.005; r = 0.71). The increase in weekly physical activity in the intervention group exceeded the minimum clinically important difference of 26 min, suggesting a significant improvement in physical activity levels.

Finally, quality of life showed improvements in the intervention group, increasing from 80.0 (95% CI: 68.8–83.8) to 85.0 (95% CI: 72.5–85.0) at follow-up, although this improvement was not significant (*p* = 0.315). In the control group, health perception decreased from 67.5 (95% CI: 32.5–78.8) to 50.0 (95% CI: 15.0–80.0), reflecting a non-significant decrease (*p* = 0.406).

In terms of feasibility outcomes, all components of the intervention, as defined in the initial protocol and detailed in Supplementary Figure 1, were delivered in full, ensuring 100% fidelity to the planned content. Adherence was monitored as the number of sessions attended in both groups. All participants attended every scheduled session, with the exception of one dropout in the control group due to work-related reasons rather than the program itself.

**Figure 3:**
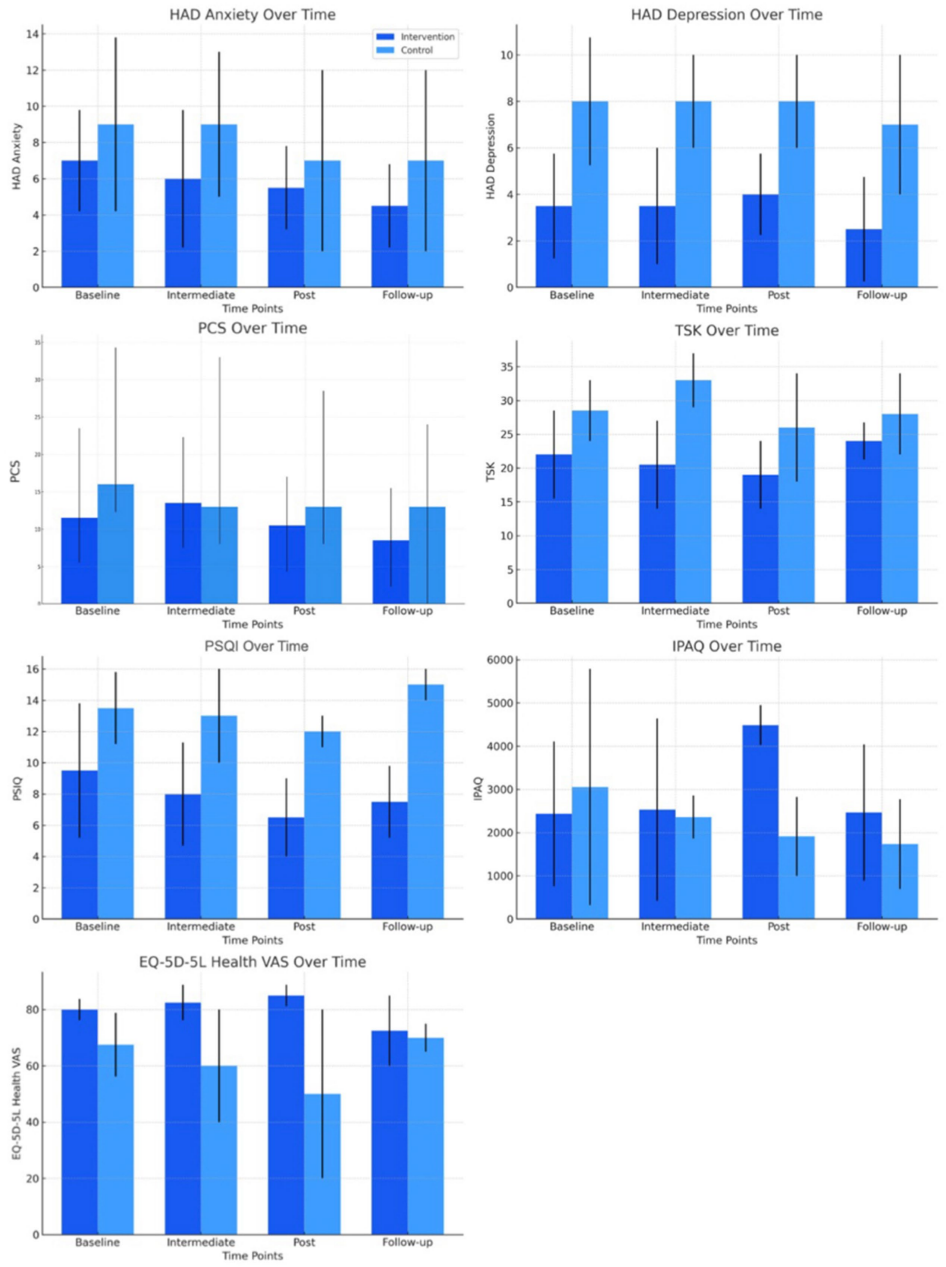
Bar plot secondary outcomes.

In summary, the results suggest that the POBTE intervention had a significant positive impact on improving certain variables such as BDNF, anxiety, depression, sleep quality, and physical activity compared to the control group (Figure 3).

### Identification of shortcomings

Throughout the study period, continuous monitoring was conducted to identify and address any emerging shortcomings that were not initially anticipated in the study protocol. The project manager engaged in ongoing communication with data collection teams, clinical personnel, and study participants. Furthermore, clinical staff were instructed to provide updates on the impact of the research on the POBTE program during regular meetings of the steering committee.

### Harms

No significant adverse effects were reported during the study. However, in session 6 out of 8, a patient from the control group experienced a fall during an exercise. After evaluation by the medical staff, it was determined that she had not sustained any relevant injuries, and the patient expressed her desire to continue with the exercise. All participants completed the sessions without any additional major incidents.

### Limitations

This study presents some limitations that should be considered when interpreting the results. The small sample size, being a pilot feasibility study, undoubtedly limits the generalizability of the clinical findings despite them being positive. For this reason, the results should be considered preliminary, and larger, adequately powered randomized controlled trials will be required to confirm these findings. Additionally, a longer follow-up period could provide a more comprehensive view of the sustainability of the observed effects, particularly regarding improvements in quality of life and other psychological variables such as depressive symptoms. An important factor to consider is the potential influence of the val66met polymorphism on BDNF levels, as this genotype may affect the secretion and transport of BDNF in the brain. Despite efforts to control external variables, such as maintaining constant circadian rhythms at the time of sample collection and ensuring a uniform temperature in the room, these measures may not have been sufficient to eliminate all sources of variability. Another limitation concerns the absence of formal correction for multiple comparisons. While this could increase the risk of Type I error (false positives), this decision was methodologically grounded in the feasibility nature of the study (Eldridge et al., 2016). Finally, we have included a population with chronic pain which is typically very heterogeneous; although these differences were considered during the sessions and pain was explained more as a central rather than peripheral phenomenon, it would be interesting to propose future studies for more specific pain groups.

### Generalizability

Despite being a pilot feasibility study, the results should be interpreted as preliminary due to the very small sample size, which substantially limits their generalizability. Nevertheless, the study shows that a low-cost, non-pharmacological intervention is feasible, well accepted, and associated with promising changes in a biomarker of cerebral plasticity (BDNF), as well as improvements in psychological variables, physical activity, and sleep problems. The high adherence and minimal dropout, with only one participant withdrawing due to work-related reasons, further reinforce the acceptability of the intervention. Moreover, the group-based format and low cost make it potentially adaptable to primary care, physiotherapy clinics, and multidisciplinary programs. Future adequately powered randomized controlled trials will be required to confirm these findings and establish their applicability to broader clinical populations.

## Discussion

The primary objective of this feasibility study was to assess the viability of conducting a randomized controlled trial of the POBTE program while examining its effects on key biomarkers of brain plasticity, such BDNF, and on pain intensity levels in individuals with chronic primary musculoskeletal pain. Additionally, this study explored various secondary variables, including anxiety, depression, sleep quality, physical activity, and general health perception, to obtain a comprehensive understanding of the intervention’s impact.

In terms of primary outcomes, BDNF levels in the intervention group exhibited a statistically significant increase from baseline to the end of treatment, remaining elevated at follow-up. In contrast, the control group maintained relatively stable levels. These results suggest that the POBTE intervention may be positively influencing neuroplasticity in these patients, inducing changes in the maladaptive plasticity typically associated with chronic pain (Costigan et al., 2009; Xiong et al., 2024). The observed increase in BDNF is consistent with previous studies where cognitive interventions, such as transcranial direct current stimulation (Podda et al., 2016; Ye et al., 2023) or the application of repetitive transcranial magnetic stimulation described by Dall’Agnol et al. (2014), have been shown to significantly elevate BDNF levels, which were associated with improvements in cognitive function. Interventions like POBTE, incorporating both cognitive and behavioral components, appear to be crucial in fostering the observed neurobiological changes. Although there is no established MCID for BDNF levels, the increases observed in the intervention group could suggest potential improvements in neuroplasticity and cognitive function, as reported in previous studies (Nilsson et al., 2020; Castaño et al., 2022; Leckie et al., 2014). Regarding pain intensity, measured by the numeric pain rating scale, the intervention group exhibited a reduction in pain scores from baseline to follow-up, although this reduction did not reach statistical significance. In contrast, the control group displayed minor fluctuations in pain levels, with no significant changes. The absence of a significant reduction in pain in the intervention group may seem surprising given the positive impact on BDNF, which is often associated with pain modulation (Merighi et al., 2008). However, it is possible that changes in BDNF reflect more medium-term neuroplastic adaptations, while pain intensity might require a longer duration or a different approach to show significant improvements. This finding aligns with some previous research suggesting that while neurobiological changes or shifts in other psychological variables may occur, they do not always directly translate into immediate pain relief (Bonatesta et al., 2022; Lluch et al., 2018), especially in populations with persistent pain where factors influencing the perception of pain intensity are multidimensional. However, the evidence regarding this variable remains unclear, as demonstrated by other studies (Siddall et al., 2022; Lin et al., 2024).

In secondary outcomes, the intervention group showed significant improvements in anxiety and depression levels. Anxiety and depression scores decreased notably in the intervention group by the end of treatment and during follow-up, unlike the control group, which showed no significant changes. These findings align with previous studies demonstrating the effectiveness of cognitive-behavioral interventions in reducing anxiety and depression in chronic pain patients (Cuenca-Martínez et al., 2023; Louw et al., 2021). The POBTE intervention, by integrating educational and coping strategies components, aids participants in better understanding and managing their condition, thereby alleviating psychological burdens. No significant changes were observed in pain catastrophizing or kinesiophobia in any group. This is consistent with research suggesting that these deeply ingrained responses may require more intensive or prolonged interventions to achieve significant changes. Specifically, Núñez-Cortés et al. (2024) in their systematic review and dose–response meta-analysis of educational models in chronic pain people, noted that altering kinesiophobia is one of the dimensions that requires the most prolonged intervention, estimating a minimum of 400 min to observe significant changes. Although health perception in the intervention group showed an improvement trend, this change was not statistically significant. This could reflect the complexity of chronic pain, where patients may require more time or more individualized interventions to perceive significant improvements in health-related quality of life (Katz, 2002). This finding aligns with the notion that while some psychological responses may be modifiable with brief interventions, others, like health perception and deeply ingrained behavioral patterns, need longer or more tailored approaches (McCracken, 2023). On the other hand, significant improvements were observed in sleep quality in the intervention group, possibly linked to reductions in anxiety and depression, and an increase in physical activity. The connection between physical activity, mental health, and sleep quality is well-documented (Scott et al., 2021; Alnawwar et al., 2023), suggesting that multi-dimensional interventions may be more effective.

## Conclusion

This feasibility pilot study indicates that the POBTE intervention is acceptable and feasible to implement, given its non-pharmacological nature and applicability in small groups across different healthcare settings. Preliminary exploratory signals are consistent with potential modulation of neuroplasticity (BDNF) and selected psychosocial outcomes (anxiety, depression, sleep quality, physical activity), supporting the biological plausibility of this approach. These findings should be interpreted with caution due to the small sample and exploratory design, and primarily serve to inform the design and justification of larger, fully powered trials.

## Data Availability

The datasets presented in this article are not readily available because access to the anonymized raw data may be considered upon reasonable request, for qualified researchers who provide a justified statement outlining the purpose and intended use of the data. Any request must include a data protection plan and will be subject to evaluation by the corresponding author and the ethics committee if necessary. Requests to access the datasets should be directed to FG-A, francisco.gurdiel@lasallecampus.es.

## Supplementary material

Supplementary Figure 1 POBTE intervention.

Supplementary Figure 2 Study process.
