# Correction: Effects of a pain oriented biobehavioral therapeutic education program on brain plasticity and pain intensity in subjects with chronic musculoskeletal pain: a feasibility study of a randomized controlled trial

**DOI:** 10.3389/fnins.2025.1756341

**Published:** 2025-12-15

**Authors:** Silvia Di-Bonaventura, Aser Donado-Bermejo, Luis Matesanz-García, Miguel Molina-Álvarez, José Vicente León-Hernández, Ángel Lizcano-Álvarez, Sergio Lerma-Lara, Maximiliano Nogales-Morales, Nuria Molina, Josué Fernández-Carnero, Francisco Gurdiel-Álvarez, Raúl Ferrer-Peña

**Affiliations:** 1 Department of Physical Therapy, Occupational Therapy, Rehabilitation and Physical Medicine, Rey Juan Carlos University, Alcorcón, Spain; 2 Escuela Internacional de Doctorado, Faculty of Health Sciences, Rey Juan Carlos University Alcorcón, Alcorcón, Spain; 3 Cognitive Neuroscience, Pain and Rehabilitation Research Group (NECODOR), Faculty of Health Sciences, Rey Juan Carlos University, Madrid, Spain; 4 Department of Physiotherapy, Centro Superior de Estudios Universitarios La Salle, Universidad Autónoma de Madrid, Madrid, Spain; 5 CranioSPain Research Group, Centro Superior de Estudios Universitarios La Salle, Universidad Autónoma de Madrid, Madrid, Spain; 6 Area of Pharmacology, Nutrition and Bromatology, Department of Basic Health Sciences, Unidad Asociada I+D+i Instituto de Química Médica (IQM) CSIC-URJC, Rey Juan Carlos University, Alcorcón, Spain; 7 Motion in Brains Research Group, Institute of Neuroscience and Sciences of the Movement (INCIMOV), Centro Superior de Estudios Universitarios La Salle, Universidad Autónoma de Madrid, Madrid, Spain; 8 Department of Nursing and Stomatology, Faculty of Health Sciences, Universidad Rey Juan Carlos, Alcorcón, Madrid, Spain; 9 Instituto de Rehabilitación Funcional La Salle, Aravaca, Calle Ganímedes, Madrid, Spain; 10 Independent Researcher, Madrid, Spain; 11 Grupo Multidisciplinar de Investigación y Tratamiento del Dolor, Grupo de Excelencia Investigadora URJC-Banco de Santander, Alcorcón, Spain; 12 La Paz Hospital Institute for Health Research, IdiPAZ, Madrid, Spain; 13 Musculoskeletal Pain and Motor Control Research Group, Faculty of Sport Sciences, Universidad Europea de Madrid, Villaviciosa de Odón, Spain; 14 Grupo de Investigación Clínico-Docente sobre Ciencias de la Rehabilitación (INDOCLIN), CSEU La Salle, UAM, Madrid, Spain

**Keywords:** pain education, brain plasticity, chronic pain, musculoskeletal pain, BDNF

Author Ángel Lizcano-Álvarez was erroneously assigned to affiliation “4 Department of Physiotherapy, Centro Superior de Estudios Universitarios La Salle, Universidad Autónoma de Madrid, Madrid, Spain”. This affiliation has now been removed for author Ángel Lizcano-Álvarez.

Affiliation “8 Department of Nursing and Stomatology, Faculty of Health Sciences, Universidad Rey Juan Carlos, Alcorcón, Madrid, Spain” was omitted from the paper. This affiliation has now been added for author Ángel Lizcano-Álvarez.

The original version of this article has been updated.

