# Supplementary figures and images for: Effects of a pain oriented biobehavioral therapeutic education program on brain plasticity and pain intensity in subjects with chronic musculoskeletal pain: a feasibility study of a randomized controlled trial
Source: Front Neurosci. 2025 Nov 17;19:1664158. doi: 10.3389/fnins.2025.1664158 (PMC12665784; doi:10.3389/fnins.2025.1664158)

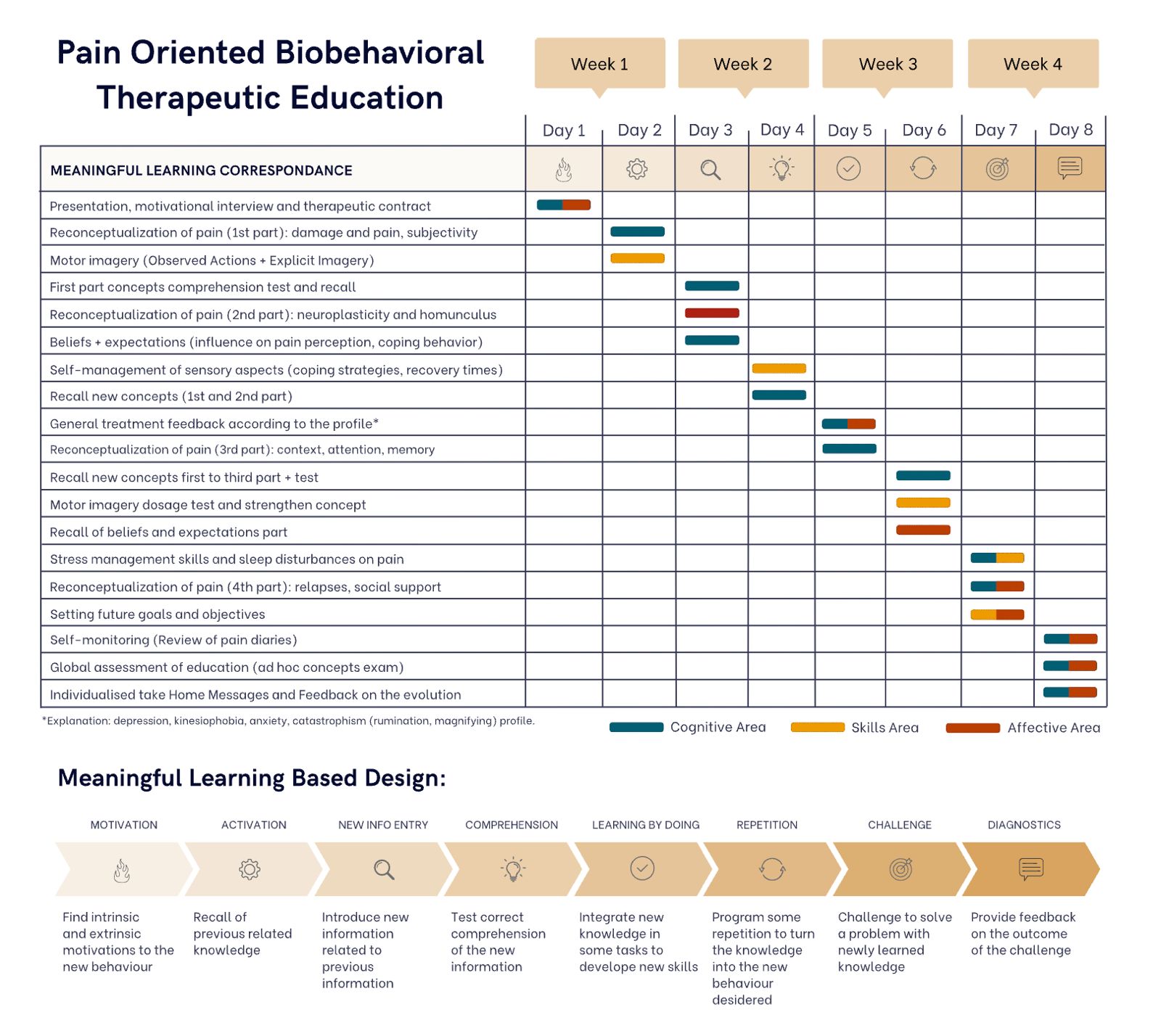

Supplement: Supplementary Figure 1 — POBTE intervention. [file Image_1.jpeg]

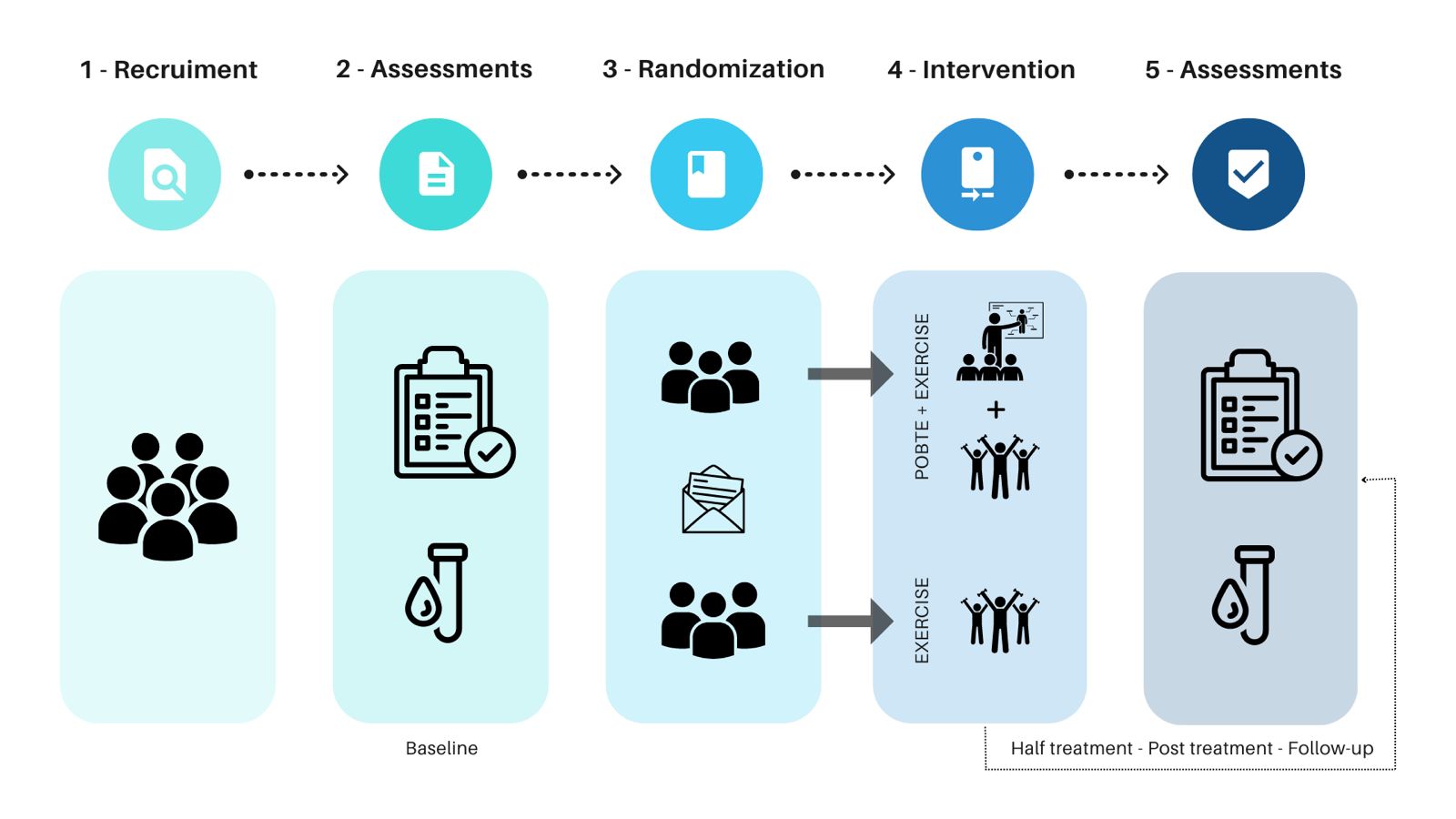

Supplement: Supplementary Figure 2 — Study process. [file Image_2.jpeg]
