# Supplementary material for: Effects of a pain oriented biobehavioral therapeutic education program on brain plasticity and pain intensity in subjects with chronic musculoskeletal pain: a feasibility study of a randomized controlled trial
Source: Front Neurosci. 2025 Nov 17;19:1664158. doi: 10.3389/fnins.2025.1664158 (PMC12665784; doi:10.3389/fnins.2025.1664158)
Supplement: Supplementary file 3 [file Data_Sheet_1.pdf]

Annex 3. Frequency of primary chronic musculoskeletal pain regions (ICD-11: chronic primary musculoskeletal pain)

Pain region	Frequency (n)	% of total sample (n=16)
Low back	11	68.8%
Lower limbs	4	25.0%
Upper limbs	3	18.8%
Dorsal	3	18.8%
Neck	2	12.5%
Participants with pain in more than one region	6	37.5%
